# Supplementary material for: Left Ventricular Noncompaction and Congenital Heart Disease Increases the Risk of Congestive Heart Failure
Source: J Clin Med. 2020 Mar 13;9(3):785. doi: 10.3390/jcm9030785 (PMC7141335; doi:10.3390/jcm9030785)
Supplement: Supplementary file 1 [file jcm-09-00785-s001.pdf]

**Supplementary Appendix**

# Left ventricular noncompaction and congenital heart disease increase risk of congestive heart failure

**Keiichi Hirono [1,*], Yukiko Hata [2], Nariaki Miyao [1], Mako Okabe [1], Shinya Takarada [1], Hideyuki Nakaoka [1], Keijiro Ibuki [1], Sayaka Ozawa [1], Naoki Yoshimura [3], Naoki Nishida [2], Fukiko Ichida [4], and LVNC study collaborators**

**<u>Table of contents:</u>**

**LVNC study collaborates.**
**Table S1. List of 182 analyzed genes of NGS.**
**Table S2. Silico predictive algorithms used in the study.**
**Table S3. List of patients with extracardiac anomaly.**
**Table S4. Gene collapsing test of rare variants.**

**LVNC study collaborates.**

Atsuhito Takeda, Atsuya Shimabukuro, Chisato Akita, Daichi Fukumi, Eiki Nishihara, Etsuko Tsuda, Heima Sakaguchi, Hidekazu Ishida, Hideshi Tomita, Hiroaki Kise, Hiroki Nagamine, Hiroki Uchiyama, Hiromi Katayama, Hiroo Ooki, Hiroshi Nishikawa, Hiroshi Ono, Hisanori Sakazaki, Hitoshi Horigome, Jun Muneuchi, Jun Yoshimoto, Junpei Soumura, Masahiro Kamada, Kazushi Yasuda, Kazuyuki Ikeda, Keiji Yasuda, Kenichi Kurosaki, Kenji Mine, Kentaro Ueno, Kiyohiro Takigiku, Kiyoshi Ogawa, Kotaro Inaguma, Kotaro Oyama, Kotaro Urayama, Kunihiko Takahashi, Kunio Ohta, Makoto Nakazawa, Mami Nakayashiro, Mamoro Ayusawa, Manatomo Toyono, Masaki Nii, Masaru Miura, Mitsuhiro Fujino, Naoshi Kuwabara, Nobuo Momoi, Nobuyuki Tsujii, Noriko Motoki, Osamu Matsuo, Reizo Baba, Ryo Inuzuka, Sachiko Kido, Satoru Iwashima, Satoshi Yasukochi, Seigo Okada, Seiichi Sato, Seki Mitsuru, Shigetoyo Kogaki, Shinsuke Hoshino, Shinya Tsukano, Shuhei Fujita, Sumito Kimura, Susumu Urata, Taichi Kato, Takako Toda, Takamichi Uchiyama, Takahiro Shindo, Takashi Higaki, Tomio Kobayashi, Tomoyasu Ozaki, Yasuhiko Tanaka, Yasuhiro Katsube, Yasunobu Hamabuchi, Yo Kajiyama, Yoko Yoshida, Yosuke Murakami, Yuriko Abe, Yoshimi Hiraumi, Yutaka Fukuda, and Yutaka Odanaka.

**Table S1. List of 182 analyzed genes of NGS.**

| Gene | Chromosome | NCBI Reference Sequence: | Sequence : (Start..End) | |
|---|---|---|---|---|
| *ABCC9* | 12p12.1 | NG_012819.1 | NC_000012.11 (21950323..22094797, complement) | http://www.ncbi.nlm.nih.gov/gene/10060 |
| *ACAD9* | 3q21.3 | NG_017064.1 | NC_000003.12 (128879490..128913114) | https://www.ncbi.nlm.nih.gov/gene/28976 |
| *ACADVL* | 17p13.1 | NG_007975.1 | NC_000017.11 (7217125..7225267) | https://www.ncbi.nlm.nih.gov/gene/37 |
| *ACTC1* | 15q14 | NG_007553.1 | NC_000015.9 (35080297..35087927, complement) | http://www.ncbi.nlm.nih.gov/gene/70 |
| *ACTN2* | 1q42-q43 | NG_009081.1 | NC_000001.10 (236849754..236927931) | http://www.ncbi.nlm.nih.gov/gene/88 |
| *ADAMTS1* | 21q21.3 | NC_000021.9 | NC_000021.9 (26836287..26845409, complement) | https://www.ncbi.nlm.nih.gov/gene/9510 |
| *ADAMTS9* | 3p14.1 | NC_000003.12 | NC_000003.12 (64515654..64688000, complement) | https://www.ncbi.nlm.nih.gov/gene/56999 |
| *ADCK3* | 1q42.13 | NG_012825.2 | NC_000001.11 (226939339..226987545) | https://www.ncbi.nlm.nih.gov/gene/56997 |
| *AKAP9* | 7q21-q22 | NG_011623.1 | NC_000007.13 (91570181..91739987 | http://www.ncbi.nlm.nih.gov/gene/10142 |
| *AMPD1* | 1p13.2 | NG_008012.1 | NC_000001.11 (114673098..114695618, complement) | https://www.ncbi.nlm.nih.gov/gene/270 |
| *ANK2* | 4q25-q27 | NG_009006.2 | NC_000004.11 (113739239..114304896) | http://www.ncbi.nlm.nih.gov/gene/287 |
| *ARFGEF2* | 20q13.13 | NG_011490.1 | NC_000020.11 (48921721..49036693) | https://www.ncbi.nlm.nih.gov/gene/10564 |
| *BAG3* | 10q25.2-q26.2 | NG_016125.1 | NC_000010.10 (121410859..121437331) | http://www.ncbi.nlm.nih.gov/gene/9531 |
| *BMP10* | 2p13.3 | NG_032117.1 | NC_000002.12 (68860916..68871517, complement) | https://www.ncbi.nlm.nih.gov/gene/27302 |
| *BMPR1A* | 10q22.3 | NG_009362.1 | NC_000010.10 (88516396..88684945) | http://www.ncbi.nlm.nih.gov/gene/657 |
| *BOLA3* | 2p13.1 | NG_031910.1 | NC_000002.12 (74135401..74147912, complement) | https://www.ncbi.nlm.nih.gov/gene/388962 |
| *BRAF* | 7q34 | NG_007873.3 | NC_000007.14 (140719327..140924928, complement) | https://www.ncbi.nlm.nih.gov/gene/673 |
| *C10orf2* | 10q24.31 | NG_012624.1 | NC_000010.11 (100987527..100994403) | https://www.ncbi.nlm.nih.gov/gene/56652 |
| *CACNA1C* | 12p13.3 | NG_008801.2 | NC_000012.11 (2079952..2807115) | http://www.ncbi.nlm.nih.gov/gene/775 |
| *CACNA2D1* | 7q21.11 | NC_000007.14 | NC_000007.14 (81946444..82443806, complement) | https://www.ncbi.nlm.nih.gov/gene/781 |
| *CACNB2* | 10p12 | NG_016195.1 | NC_000010.10 (18429373..18830688) | http://www.ncbi.nlm.nih.gov/gene/783 |
| *CALR3* | 19p13.11 | NG_031959.2 | NC_000019.9 (16589767..16607015, complement) | http://www.ncbi.nlm.nih.gov/gene/125972 |
| *CAPN3* | 15q15.1 | NG_008660.1 | NC_000015.9 (42646545..42704515) | http://www.ncbi.nlm.nih.gov/gene/825 |
| *CASQ2* | 1p13.1 | NG_008802.1 | NC_000001.11 (115700003..115768805, complement) | https://www.ncbi.nlm.nih.gov/gene/845 |

| | | | | |
|---|---|---|---|---|
| *CASZ1* | 1p36.22 | NC_000001.11 | NC_000001.11 (10636604..10796676, complement) | https://www.ncbi.nlm.nih.gov/gene/54897 |
| *CAV3* | 3p25 | NG_008797.2 | NC_000003.11 (8775486..8788451) | http://www.ncbi.nlm.nih.gov/gene/859 |
| *CDKN1C* | 11p15.4 | NG_008022.1 | NC_000011.10 (2883218..2885804, complement) | https://www.ncbi.nlm.nih.gov/gene/1028 |
| *COL4A1* | 13q34 | NG_011544.1 | NC_000013.10 (110801310..110959496, complement) | http://www.ncbi.nlm.nih.gov/gene/1282 |
| *COL7A1* | 3p21.31 | NG_007065.1 | NC_000003.12 (48564073..48595302, complement) | https://www.ncbi.nlm.nih.gov/gene/1294 |
| *CPT2* | 1p32.3 | NG_008035.1 | NC_000001.11 (53196429..53214197) | https://www.ncbi.nlm.nih.gov/gene/1376 |
| *CSRP3* | 11p15.1 | NG_011932.2 | NC_000011.10 (19182030..19210571, complement) | https://www.ncbi.nlm.nih.gov/gene/8048 |
| *CTNNA3* | 10q21.3 | NG_034072.1 | NC_000010.11 (65912518..67696217, complement) | https://www.ncbi.nlm.nih.gov/gene/29119 |
| *DAAM1* | 14q23.1 | NG_047127.1 | NC_000014.9 (59188657..59371405) | https://www.ncbi.nlm.nih.gov/gene/23002 |
| *DAAM2* | 6p21.2 | NC_000006.12 | NC_000006.12 (39792366..39904877) | https://www.ncbi.nlm.nih.gov/gene/23500 |
| *DES* | 2q35 | NG_008043.1 | NC_000002.11 (220283099..220291461) | http://www.ncbi.nlm.nih.gov/gene/1674 |
| *DMD* | Xp21.2 | NG_012232.1 | NC_000023.10 (31137345..33357726, complement) | http://www.ncbi.nlm.nih.gov/gene/1756 |
| *DMPK* | 19q13.32 | NG_009784.1 | NC_000019.10 (45769709..45782557, complement) | https://www.ncbi.nlm.nih.gov/gene/1760 |
| *DNAJC19* | 3q26.33 | NG_022933.1 | NC_000003.12 (180983709..180989774, complement) | https://www.ncbi.nlm.nih.gov/gene/131118 |
| *DSC2* | 18q12.1 | NG_008208.1 | NC_000018.9 (28645938..28682388, complement) | http://www.ncbi.nlm.nih.gov/gene/1824 |
| *DSG2* | 18q12.1 | NG_007072.3 | NC_000018.9 (29078027..29128814) | http://www.ncbi.nlm.nih.gov/gene/1829 |
| *DSP* | 6p24 | NG_008803.1 | NC_000006.11 (7541808..7586946) | http://www.ncbi.nlm.nih.gov/gene/1832 |
| *DTNA* | 18q12.1 | NG_009201.1 | NC_000018.10 (34493290..34891844) | https://www.ncbi.nlm.nih.gov/gene/1837 |
| *DVL1* | 1p36.33 | NG_008048.1 | NC_000001.11 (1335278..1349142, complement) | https://www.ncbi.nlm.nih.gov/gene/1855 |
| *EED* | 11q14.2 | NG_029595.1 | NC_000011.10 (86244384..86285420) | https://www.ncbi.nlm.nih.gov/gene/8726 |
| *ELN* | 7q11.23 | NG_009261.1 | NC_000007.13 (73442119..73484237) | http://www.ncbi.nlm.nih.gov/gene/2006 |
| *EMD* | Xq28 | NG_008677.1 | NC_000023.10 (153607597..153609883) | http://www.ncbi.nlm.nih.gov/gene/2010 |
| *ERBB2* | 17q12 | NG_007503.1 | NC_000017.11 (39688084..39728662) | https://www.ncbi.nlm.nih.gov/gene/2064 |
| *ERBB4* | 2q34 | NG_011805.1 | NC_000002.12 (211375717..212538628, complement) | https://www.ncbi.nlm.nih.gov/gene/2066 |
| *EZH2* | 7q36.1 | NG_032043.1 | NC_000007.14 (148807372..148884349, complement) | https://www.ncbi.nlm.nih.gov/gene/2146 |
| *FBN2* | 5q23.3 | NG_008750.1 | NC_000005.10 (128257909..128538042, complement) | https://www.ncbi.nlm.nih.gov/gene/2201 |

| | | | | |
|---|---|---|---|---|
| *FGF16* | Xq21.1 | NG_034050.1 | NC_000023.11 (77447675..77456522) | https://www.ncbi.nlm.nih.gov/gene/8823 |
| *FGF9* | 13q12.11 | NG_016272.1 | NC_000013.11 (21671076..21704501) | https://www.ncbi.nlm.nih.gov/gene/2254 |
| *FGFR1* | 8p11.23 | NG_007729.1 | NC_000008.11 (38411138..38468834, complement) | https://www.ncbi.nlm.nih.gov/gene/2260 |
| *FGFR2* | 10q26.13 | NG_012449.2 | NC_000010.11 (121478330..121598458, complement) | https://www.ncbi.nlm.nih.gov/gene/2263 |
| *FKBP1A* | 20p13 | NC_000020.11 | NC_000020.11 (1368977..1393172, complement) | https://www.ncbi.nlm.nih.gov/gene/2280 |
| *FKBP1B* | 2p23.3 | NC_000002.12 | NC_000002.12 (24033205..24067743) | https://www.ncbi.nlm.nih.gov/gene/2281 |
| *FKTN* | 9q31.2 | NG_008754.1 | NC_000009.12 (105558117..105655950) | https://www.ncbi.nlm.nih.gov/gene/2218 |
| *FLNA* | Xq28 | NG_011506.1 | NC_000023.11 (154348532..154374638, complement) | https://www.ncbi.nlm.nih.gov/gene/2316 |
| *FXN* | 9q21.11 | NG_008845.2 | NC_000009.12 (69035563..69079077) | https://www.ncbi.nlm.nih.gov/gene/2395 |
| *GAA* | 17q25.2-q25.3 | NG_009822.1 | NC_000017.10 (78075339..78093680) | http://www.ncbi.nlm.nih.gov/gene/2548 |
| *GATA4* | 8p23.1-p22 | NG_008177.1 | NC_000008.10 (11534433..11617510) | http://www.ncbi.nlm.nih.gov/gene/2626 |
| *GBE1* | 3p12.2 | NG_011810.1 | NC_000003.12 (81489699..81761799, complement) | https://www.ncbi.nlm.nih.gov/gene/2632 |
| *GFRA1* | 10q25.3 | NG_050620.1 | NC_000010.11 (116056925..116273645, complement) | https://www.ncbi.nlm.nih.gov/gene/2674 |
| *GFRA2* | 8p21.3 | NG_029215.1 | NC_000008.11 (21690403..21789296, complement) | https://www.ncbi.nlm.nih.gov/gene/2675 |
| *GLA* | Xq22 | NG_007119.1 | NC_000023.10 (100652779..100663001, complement) | http://www.ncbi.nlm.nih.gov/gene/2717 |
| *GPD1L* | 3p22.3 | NG_023375.1 | NC_000003.11 (32148003..32210207) | http://www.ncbi.nlm.nih.gov/gene/23171 |
| *HADHA* | 2p23.3 | NG_007121.1 | NC_000002.12 (26190635..26244726, complement) | https://www.ncbi.nlm.nih.gov/gene/3030 |
| *HAS2* | 8q24.13 | NC_000008.11 | NC_000008.11 (121613031..121641390, complement) | https://www.ncbi.nlm.nih.gov/gene/3037 |
| *HBB* | 11p15.4 | NG_059281.1 | NC_000011.10 (5225466..5227071, complement) | https://www.ncbi.nlm.nih.gov/gene/3043 |
| *HCCS* | Xp22.2 | NG_016460.1 | NC_000023.11 (11111286..11123086) | https://www.ncbi.nlm.nih.gov/gene/3052 |
| *HCN4* | 15q24.1 | NG_009063.1 | NC_000015.9 (73612200..73661605, complement) | http://www.ncbi.nlm.nih.gov/gene/10021 |
| *HEY2* | 6q22.31 | NC_000006.12 | NC_000006.12 (125747639..125762243) | https://www.ncbi.nlm.nih.gov/gene/23493 |
| *HMGCL* | 1p36.11 | NG_013061.1 | NC_000001.11 (23801877..23825459, complement) | https://www.ncbi.nlm.nih.gov/gene/3155 |
| *ITGA7* | 12q13.2 | NG_012343.1 | NC_000012.12 (55684568..55716037, complement) | https://www.ncbi.nlm.nih.gov/gene/3679 |
| *JARID2* | 6p22.3 | NC_000006.12 | NC_000006.12 (15245975..15522042) | https://www.ncbi.nlm.nih.gov/gene/3720 |
| *JUP* | 17q21 | NG_009090.2 | NC_000017.10 (39910859..39942964, complement) | http://www.ncbi.nlm.nih.gov/gene/3728 |

| | | | | |
|---|---|---|---|---|
| *KCNE1* | 21q22.12 | NG_009091.1 | NC_000021.8 (35790910..35884573, complement) | http://www.ncbi.nlm.nih.gov/gene/3753 |
| *KCNE2* | 21q22.12 | NG_008804.1 | NC_000021.8 (35736323..35743440) | http://www.ncbi.nlm.nih.gov/gene/9992 |
| *KCNE3* | 11q13.4 | NG_011833.1 | NC_000011.9 (74165886..74178600, complement) | http://www.ncbi.nlm.nih.gov/gene/10008 |
| *KCNH2* | 7q36.1 | NG_008916.1 | NC_000007.13 (150642044..150675402, complement) | http://www.ncbi.nlm.nih.gov/gene/3757 |
| *KCNJ2* | 17q24.3 | NG_008798.1 | NC_000017.10 (68164757..68176189) | http://www.ncbi.nlm.nih.gov/gene/3759 |
| *KCNQ1* | 11p15.5 | NG_008935.1 | NC_000011.9 (2466221..2870340) | http://www.ncbi.nlm.nih.gov/gene/3784 |
| *KRAS* | 12p12.1 | NG_007524.1 | NC_000012.11 (25358180..25403870, complement) | http://www.ncbi.nlm.nih.gov/gene/3845 |
| *LAMP2* | Xq24 | NG_007995.1 | NC_000023.10 (119560003..119603204, complement) | http://www.ncbi.nlm.nih.gov/gene/3920 |
| *LDB3* | 10q22.3-q23.2 | NG_008876.1 | NC_000010.10 (88426542..88495829) | http://www.ncbi.nlm.nih.gov/gene/11155 |
| *LMNA* | 1q22 | NG_008692.2 | NC_000001.10 (156052369..156109880) | http://www.ncbi.nlm.nih.gov/gene/4000 |
| *LMX1B* | 9q33.3 | NG_017039.1 | NC_000009.12 (126614443..126701032) | https://www.ncbi.nlm.nih.gov/gene/4010 |
| *LRPPRC* | 2p21 | NG_008247.1 | NC_000002.12 (43886224..43996005, complement) | https://www.ncbi.nlm.nih.gov/gene/10128 |
| *MADD* | 11p11.2 | NG_029462.1 | NC_000011.10 (47269376..47330031) | https://www.ncbi.nlm.nih.gov/gene/8567 |
| *MBL2* | 10q21.1 | NG_008196.1 | NC_000010.11 (52764977..52772847, complement) | https://www.ncbi.nlm.nih.gov/gene/4153 |
| *MED1* | 17q12 | NG_046996.1 | NC_000017.11 (39404285..39451281, complement) | https://www.ncbi.nlm.nih.gov/gene/5469 |
| *MEST* | 7q32.2 | NG_009226.1 | NC_000007.14 (130486175..130506297) | https://www.ncbi.nlm.nih.gov/gene/4232 |
| *MIB1* | 18q11.2 | NG_033272.2 | NC_000018.10 (21740793..21870957) | https://www.ncbi.nlm.nih.gov/gene/57534 |
| *MIPEP* | 13q12.12 | NG_052977.1 | NC_000013.11 (23730189..23889448, complement) | https://www.ncbi.nlm.nih.gov/gene/4285 |
| *MLYCD* | 16q23.3 | NG_009079.1 | NC_000016.10 (83899125..83916182) | https://www.ncbi.nlm.nih.gov/gene/23417 |
| *MMACHC* | 1p34.1 | NG_013378.1 | NC_000001.11 (45500184..45511266) | https://www.ncbi.nlm.nih.gov/gene/25974 |
| *MRPS22* | 3q23 | NG_012174.1 | NC_000003.12 (139344014..139357129) | https://www.ncbi.nlm.nih.gov/gene/56945 |
| *MTO1* | 6q13 | NG_032856.1 | NC_000006.12 (73461731..73501456) | https://www.ncbi.nlm.nih.gov/gene/25821 |
| *MYBPC3* | 11p11.2 | NG_007667.1 | NC_000011.9 (47352957..47374253, complement) | http://www.ncbi.nlm.nih.gov/gene/4607 |
| *MYCN* | 2p24.3 | NG_007457.1 | NC_000002.12 (15940438..15947007) | https://www.ncbi.nlm.nih.gov/gene/4613 |
| *MYH11* | 16p13.11 | NG_009299.1 | NC_000016.9 (15796992..15950887, complement) | http://www.ncbi.nlm.nih.gov/gene/4629 |
| *MYH6* | 14q12 | NG_023444.1 | NC_000014.8 (23849942..23878836, complement) | http://www.ncbi.nlm.nih.gov/gene/4624 |
| *MYH7* | 14q12 | NG_007884.1 | NC_000014.8 (23881947..23904870, complement) | http://www.ncbi.nlm.nih.gov/gene/4625 |
| *MYH7B* | 20q11.22 | NG_016984.2 | NC_000020.11 (34955835..35002437) | https://www.ncbi.nlm.nih.gov/gene/57644 |

| | | | | |
|---|---|---|---|---|
| *MYL2* | 12q24.11 | NG_007554.1 | NC_000012.11 (111348623..111358404, complement) | http://www.ncbi.nlm.nih.gov/gene/4633 |
| *MYL3* | 3p21.3-p21.2 | NG_007555.2 | NC_000003.11 (46899357..46904973, complement) | http://www.ncbi.nlm.nih.gov/gene/4634 |
| *MYLK* | 3q21 | NG_029111.1 | NC_000003.11 (123331143..123603149, complement) | http://www.ncbi.nlm.nih.gov/gene/4638 |
| *MYOZ2* | 4q26-q27 | NG_029747.1 | NC_000004.11 (120056939..120108944) | http://www.ncbi.nlm.nih.gov/gene/51778 |
| *NEXN* | 1p31.1 | NG_016625.1 | NC_000001.11 (77888515..77948643) | https://www.ncbi.nlm.nih.gov/gene/91624 |
| *NFATC1* | 18q23 | NG_029226.1 | NC_000018.10 (79395772..79529323) | https://www.ncbi.nlm.nih.gov/gene/4772 |
| *NKX2-5* | 5q34 | NG_013340.1 | NC_000005.9 (172659107..172662315, complement) | http://www.ncbi.nlm.nih.gov/gene/1482 |
| *NNT* | 5p12 | NG_032869.1 | NC_000005.10 (43601092..43705566) | https://www.ncbi.nlm.nih.gov/gene/23530 |
| *NR0B1* | Xp21.2 | NG_009814.1 | NC_000023.11 (30304422..30309378, complement) | https://www.ncbi.nlm.nih.gov/gene/190 |
| *NRAS* | 1p13.2 | NG_007572.1 | NC_000001.10 (115247085..115259515, complement) | http://www.ncbi.nlm.nih.gov/gene/4893 |
| *NRG1* | 8p12 | NG_012005.2 | NC_000008.11 (31639222..32771716) | https://www.ncbi.nlm.nih.gov/gene/3084 |
| *NSD1* | 5q35.3 | NG_009821.1 | NC_000005.10 (177131835..177300213) | https://www.ncbi.nlm.nih.gov/gene/64324 |
| *NUMB* | 14q24.2-q24.3 | NG_029061.2 | NC_000014.9 (73275210..73458580, complement) | https://www.ncbi.nlm.nih.gov/gene/8650 |
| *NUMBL* | 19q13.2 | NC_000019.10 | NC_000019.10 (40665905..40690658, complement) | https://www.ncbi.nlm.nih.gov/gene/9253 |
| *PKP2* | 12p11 | NG_009000.1 | NC_000012.11 (32943680..33049780, complement) | http://www.ncbi.nlm.nih.gov/gene/5318 |
| *PLEC* | 8q24.3 | NG_012492.1 | NC_000008.11 (143915147..143976800, complement) | https://www.ncbi.nlm.nih.gov/gene/5339 |
| *PLEKHM2* | 1p36.21 | NG_053033.1 | NC_000001.11 (15681506..15734769) | https://www.ncbi.nlm.nih.gov/gene/23207 |
| *PLN* | 6q22.1 | NG_009082.1 | NC_000006.11 (118869442..118881587) | http://www.ncbi.nlm.nih.gov/gene/5350 |
| *PMP22* | 17p12 | NG_007949.1 | NC_000017.11 (15229777..15265373, complement) | https://www.ncbi.nlm.nih.gov/gene/5376 |
| *POLG* | 15q26.1 | NG_008218.2 | NC_000015.10 (89316305..89334795, complement) | https://www.ncbi.nlm.nih.gov/gene/5428 |
| *PRDM16* | 1p36.32 | NG_029576.1 | NC_000001.11 (3068227..3438621) | https://www.ncbi.nlm.nih.gov/gene/63976 |
| *PRKAG2* | 7q36.1 | NG_007486.1 | NC_000007.13 (151253200..151574316, complement) | http://www.ncbi.nlm.nih.gov/gene/51422 |
| *PTGS2* | 1q31.1 | NG_028206.2 | NC_000001.11 (186671812..186680427, complement) | https://www.ncbi.nlm.nih.gov/gene/5743 |
| *PTK2* | 8q24.3 | NG_029467.1 | NC_000008.11 (140658382..141002079, complement) | https://www.ncbi.nlm.nih.gov/gene/5747 |

| | | | | |
|---|---|---|---|---|
| *PTPN11* | 12q24 | NG_007459.1 | NC_000012.11 (112856536..112947717) | http://www.ncbi.nlm.nih.gov/gene/5781 |
| *RAD54L2* | 3p21.2 | NC_000003.12 | NC_000003.12 (51538683..51668660) | https://www.ncbi.nlm.nih.gov/gene/23132 |
| *RAF1* | 3p25 | NG_007467.1 | NC_000003.11 (12625100..12705700, complement) | http://www.ncbi.nlm.nih.gov/gene/5894 |
| *RANGRF* | 17p13.1 | NG_028189.1 | NC_000017.11 (8288497..8290092) | https://www.ncbi.nlm.nih.gov/gene/29098 |
| *RBM20* | 10q25.2 | NG_021177.1 | NC_000010.11 (110641933..110839471) | https://www.ncbi.nlm.nih.gov/gene/282996 |
| *RIT1* | 1q22 | NG_033885.1 | NC_000001.11 (155897808..155911402, complement) | https://www.ncbi.nlm.nih.gov/gene/6016 |
| *RPS6KA3* | Xp22.12 | NG_007488.1 | NC_000023.11 (20149911..20267514, complement) | https://www.ncbi.nlm.nih.gov/gene/6197 |
| *RPS7* | 2p25 | NG_011744.1 | NC_000002.11 (3622853..3628509) | http://www.ncbi.nlm.nih.gov/gene/6201 |
| *RYR1* | 19q13.2 | NG_008866.1 | NC_000019.10 (38433700..38587564) | https://www.ncbi.nlm.nih.gov/gene/6261 |
| *RYR2* | 1q43 | NG_008799.2 | NC_000001.10 (237205510..237997288) | http://www.ncbi.nlm.nih.gov/gene/6262 |
| *SCN1B* | 9q13.1 | NG_013359.1 | NC_000019.9 (35521555..35531353) | http://www.ncbi.nlm.nih.gov/gene/6324 |
| *SCN3B* | 11q23.3 | NG_016283.1 | NC_000011.9 (123499895..123525315, complement) | http://www.ncbi.nlm.nih.gov/gene/55800 |
| *SCN4B* | 11q23.3 | NG_011710.1 | NC_000011.9 (118004092..118023630, complement) | http://www.ncbi.nlm.nih.gov/gene/6330 |
| *SCN5A* | 3p21 | NG_008934.1 | NC_000003.11 (38589553..38691164, complement) | http://www.ncbi.nlm.nih.gov/gene/6331 |
| *SCO2* | 22q13.33 | NG_016235.1 | NC_000022.11 (50523568..50526439, complement) | https://www.ncbi.nlm.nih.gov/gene/9997 |
| *SCRIB* | 8q24.3 | NG_030583.1 | NC_000008.11 (143790920..143815379, complement) | https://www.ncbi.nlm.nih.gov/gene/23513 |
| *SDHA* | 5p15.33 | NG_012339.1 | NC_000005.10 (218223..264816) | https://www.ncbi.nlm.nih.gov/gene/6389 |
| *SDHAF1* | 19q13.12 | NG_016869.1 | NC_000019.10 (35995188..35996318) | https://www.ncbi.nlm.nih.gov/gene/644096 |
| *SDHB* | 1p36.13 | NG_012340.1 | NC_000001.11 (17018722..17054170, complement) | https://www.ncbi.nlm.nih.gov/gene/6390 |
| *SDS* | 12q24.13 | NC_000012.12 | NC_000012.12 (113392445..113403887, complement) | https://www.ncbi.nlm.nih.gov/gene/10993 |
| *SGCD* | 5q33-q34 | NG_008693.2 | NC_000005.9 (155462147..156194799) | http://www.ncbi.nlm.nih.gov/gene/6444 |
| *SLC22A5* | 5q31.1 | NG_008982.2 | NC_000005.10 (132369704..132395614) | https://www.ncbi.nlm.nih.gov/gene/6584 |
| *SLC25A20* | 3p21.31 | NG_008171.1 | NC_000003.12 (48856923..48898993, complement) | https://www.ncbi.nlm.nih.gov/gene/788 |
| *SLC25A4* | 4q35 | NG_013001.1 | NC_000004.11 (186064417..186071538) | http://www.ncbi.nlm.nih.gov/gene/291 |
| *SLC25A4* | 4q35.1 | NG_013001.1 | NC_000004.12 (185143263..185150384) | https://www.ncbi.nlm.nih.gov/gene/291 |
| *SLC25A5* | Xq24 | NG_013262.1 | NC_000023.11 (119468400..119471396) | https://www.ncbi.nlm.nih.gov/gene/292 |
| *SLC52A2* | 8q24.3 | NG_032872.1 | NC_000008.11 (144358547..144361286) | https://www.ncbi.nlm.nih.gov/gene/79581 |
| *SMAD3* | 15q22.33 | NG_011990.1 | NC_000015.9 (67358036..67487533) | http://www.ncbi.nlm.nih.gov/gene/4088 |

| | | | | |
|---|---|---|---|---|
| *SMAD7* | 18q21.1 | NG_023330.1 | NC_000018.10 (48919853..48950711, complement) | https://www.ncbi.nlm.nih.gov/gene/4092 |
| *SMARCA4* | 19p13.2 | NG_011556.2 | NC_000019.10 (10960922..11062282) | https://www.ncbi.nlm.nih.gov/gene/6597 |
| *SNTA1* | 20q11.2 | NG_011622.1 | NC_000020.10 (31995763..32031698, complement) | http://www.ncbi.nlm.nih.gov/gene/6640 |
| *SOS1* | 2p21 | NG_007530.1 | NC_000002.11 (39208690..39347686, complement) | http://www.ncbi.nlm.nih.gov/gene/6654 |
| *SQSTM1* | 5q35.3 | NG_011342.1 | NC_000005.10 (179806388..179838078) | https://www.ncbi.nlm.nih.gov/gene/8878 |
| *STARD3* | 17q11-q12 | | NC_000017.10 (37793333..37820454) | http://www.ncbi.nlm.nih.gov/gene/10948 |
| *TAZ* | Xq28 | NG_009634.1 | NC_000023.10 (153639877..153650065) | http://www.ncbi.nlm.nih.gov/gene/6901 |
| *TBX20* | 7p14.2 | NG_015805.1 | NC_000007.14 (35199936..35254100, complement) | https://www.ncbi.nlm.nih.gov/gene/57057 |
| *TBX5* | 12q24.1 | NG_007373.1 | NC_000012.11 (114791735..114846247, complement) | http://www.ncbi.nlm.nih.gov/gene/6910 |
| *TCAP* | 17q12 | NG_008892.1 | NC_000017.11 (39665346..39666554) | https://www.ncbi.nlm.nih.gov/gene/8557 |
| *TGFB3* | 14q24.3 | NG_011715.1 | NC_000014.9 (75958061..75983011, complement) | https://www.ncbi.nlm.nih.gov/gene/7043 |
| *TGFBR1* | 9q22 | NG_007461.1 | NC_000009.11 (101867412..101916474) | http://www.ncbi.nlm.nih.gov/gene/7046 |
| *TGFBR2* | 3p22 | NG_007490.1 | NC_000003.11 (30647994..30735634) | http://www.ncbi.nlm.nih.gov/gene/7048 |
| *TMEM43* | 3p25.1 | NG_008975.1 | NC_000003.11 (14166440..14185180) | http://www.ncbi.nlm.nih.gov/gene/79188 |
| *TMEM70* | 8q21.11 | NG_016618.1 | NC_000008.11 (73976142..73982783) | https://www.ncbi.nlm.nih.gov/gene/54968 |
| *TNNC1* | 3p21.1 | NG_008963.1 | NC_000003.11 (52485107..52488057, complement) | http://www.ncbi.nlm.nih.gov/gene/7134 |
| *TNNI3* | 19q13.4 | NG_007866.2 | NC_000019.9 (55663135..55669100, complement) | http://www.ncbi.nlm.nih.gov/gene/7137 |
| *TNNT2* | 1q32 | NG_007556.1 | NC_000001.10 (201328136..201346836, complement) | http://www.ncbi.nlm.nih.gov/gene/7139 |
| *TPM1* | 15q22.1 | NG_007557.1 | NC_000015.9 (63334838..63364114) | http://www.ncbi.nlm.nih.gov/gene/7168 |
| *TTN* | 2q31.2 | NG_011618.3 | NC_000002.12 (178525989..178807423, complement) | https://www.ncbi.nlm.nih.gov/gene/7273 |
| *TTR* | 18q12.1 | NG_009490.1 | NC_000018.10 (31591767..31599024) | https://www.ncbi.nlm.nih.gov/gene/7276 |
| *VANGL2* | 1q23.2 | NG_023420.1 | NC_000001.11 (160400574..160428678) | https://www.ncbi.nlm.nih.gov/gene/57216 |
| *VCL* | 10q22.2 | NG_008868.1 | NC_000010.10 (75757836..75879918) | http://www.ncbi.nlm.nih.gov/gene/7414 |
| *YAP1* | 11q22.1 | NG_029530.1 | NC_000011.10 (102109957..102233423) | https://www.ncbi.nlm.nih.gov/gene/10413 |
| *YWHAE* | 17p13.3 | NG_009233.1 | NC_000017.11 (1344539..1400262, complement) | https://www.ncbi.nlm.nih.gov/gene/7531 |
| *ZNF25* | 10p11.21 | NC_000010.11 | NC_000010.11 (37949572..37976655, complement) | https://www.ncbi.nlm.nih.gov/gene/219749 |

**Table S2. Silico predictive algorithms used in the study.**

| Category | Basis | Name | Website | Prediction Threshold |
|---|---|---|---|---|
| Missense prediction | Evolutionary conservation | FATHMM | http://fathmm.biocompute.org.uk | <-1.5 Damaging<br>>-1.5 Tolerated |
| | | SIFT | http://sift.jcvi.org | <0.05 Deleterious<br>>0.05 Tolerated |
| Missense prediction | Protein structure/function and evolutionary conservation | Align GVGD | http://agvgd.iarc.fr/agvgd_input.php | ≧C15 Probably Damaging |
| | | Mutation Taster | http://www.mutationtaster.org | Disease causing |
| | | Polyphen-2 | http://genetics.bwh.harvard.edu/pph2 | 0.85 to 1 Probably Damage<br>0.15 to 0.85 Possibly Damage |
| Missense and insertion/deletions prediction | Alignment and measurement of similarity between variant sequence and protein sequence homolog | PROVEAN | http://provean.jcvi.org/index.php | <-2.5 Deleterious<br>>-2.5 Neutral |
| Missense and insertion/deletions prediction | Contrasts annotations of fixed/nearly fixed derived alleles in humans with simulated variants | CADD | http://cadd.gs.washington.edu | ≧20 1% most deleterious<br>≧30 0.1%most deleterious |

Reference

1.Richards S, Aziz N, Bale S, Bick D, Das S, Gastier-Foster J, Grody WW, Hegde M, Lyon E, Spector E, Voelkerding K, Rehm HL. Standards and guidelines for the interpretation of sequence variants: a joint consensus recommendation of the American College of Medical Genetics and Genomics and the association for molecular autopsy. Genet Med 2015;17:405–423.

**Table S3. List of patients with extracardiac anomaly.**

| Disease | Number of patients |
|---|---|
| Motor development delay | 18 |
| Mental retardation | 15 |
| Leigh encephalopathy | 2 |
| Odd looking face | 2 |
| Blepharophimosis, micrognathia | 1 |
| Corpus callosum hypoplasia | 1 |
| Corpus callosum defect, primary vitreous hyperplasia remnant | 1 |
| Deafness, umbilical hernia, high arched palate, muscular hypertonia | 1 |
| Eye corneal opacity, cornea rear deficit, small eyeballs | 1 |
| Forehead protruding | 1 |
| Forehead protruding, micrognathia | 1 |
| Forehead protruding, high arched palate | 1 |
| Glycogen disease | 1 |
| High arched palate, arthrogryposis, muscular hypotonia | 1 |
| Macrocephaly | 1 |
| Microcephaly | 1 |
| Mitochondrial disease | 1 |
| Muscular hypotonia, epilepsy | 1 |
| Labia majora hypertrophy, corneal opacity | 1 |
| Nephrotic syndrome, macrocephaly | 1 |
| Polycystic kidney | 1 |
| Proptosis, low set ear, high arched palate | 1 |
| Smooth encephalopathy | 1 |
| Slanted eyebrows, slanted palpebral fissure | 1 |
| Symphalangism, pyloric stenosis | 1 |

**Table S4. Gene collapsing test of rare variants.**

| Rank | Gene | Qualifying Cases | Frequency Qualifying Cases (n=53) | Qualifying Controls Cases | Frequency Qualifying Controls (n=4456) | Fisher's Exact Test p-value |
|---|---|---|---|---|---|---|
| 1 | *MYH7* | 8 | 0.1509 | 0 | 0 | $2.104 \times 10^{-16}$ |
| 2 | *TPM1* | 2 | 0.0377 | 0 | 0 | $1.356 \times 10^{-4}$ |
| 3 | *ACTC1* | 1 | 0.0189 | 0 | 0 | 0.0119 |
| 3 | *ANK2* | 1 | 0.0189 | 2 | 0.0004 | 0.0349 |
| 3 | *COL4A1* | 1 | 0.0189 | 2 | 0.0004 | 0.0349 |
| 3 | *DAAM1* | 1 | 0.0189 | 0 | 0 | 0.0119 |
| 3 | *DSG2* | 1 | 0.0189 | 0 | 0 | 0.0119 |
| 3 | *DSP* | 1 | 0.0189 | 0 | 0 | 0.0119 |
| 3 | *FGF16* | 1 | 0.0189 | 0 | 0 | 0.0119 |
| 3 | *FGFR2* | 1 | 0.0189 | 0 | 0 | 0.0119 |
| 3 | *HCN4* | 1 | 0.0189 | 0 | 0 | 0.0119 |
| 3 | *JUP* | 1 | 0.0189 | 0 | 0 | 0.0119 |
| 3 | *MYBPC3* | 1 | 0.0189 | 0 | 0 | 0.0119 |
| 3 | *MYH6* | 1 | 0.0189 | 0 | 0 | 0.0119 |
| 3 | *MYL2* | 1 | 0.0189 | 0 | 0 | 0.0119 |
| 3 | *PKP2* | 1 | 0.0189 | 0 | 0 | 0.0119 |
| 3 | *PRDM16* | 1 | 0.0189 | 0 | 0 | 0.0119 |
| 3 | *RYR2* | 1 | 0.0189 | 0 | 0 | 0.0119 |
